# Odonate Diversity Patterns in Italy Disclose Intricate Colonization Pathways

**DOI:** 10.3390/biology11060886

**Published:** 2022-06-08

**Authors:** Simone Fattorini

**Affiliations:** Department of Life, Health and Environmental Sciences, University of L’Aquila, 67100 L’Aquila, Italy; simone.fattorini@univaq.it

**Keywords:** beta diversity, biogeography, dragonflies, glacial refugia, ice age, macroecology, Mediterranean, Odonata, peninsula effect, Pleistocene

## Abstract

**Simple Summary:**

During the last Ice Age, most European animals retreated into southern refuges (mainly the Iberian, Italian, and Balkan peninsulas) from which they recolonized central and northern countries after deglaciation. These medio-European territories may have subsequently acted as secondary centers of southward dispersion for many species. Acting both as a refuge and as an area of colonization from adjacent territories, Italy was the theater of complex biogeographical histories, as illustrated by current distributional patterns of odonates (damselflies and dragonflies). These patterns are a result of historical factors and current ecological conditions. Odonates need freshwater for their development, and their richness in Italy decreases southwards, both because of a decrease in precipitation and because of increasing distance from the mainland (peninsula effect). Biogeographical composition of Italian regions is influenced by climate, geographical distances and historical factors. In particular, biogeographical similarities between Italian regions and adjacent areas revealed multiple colonization patterns. After serving as a glacial refuge from which odonates may have colonized medio-European areas, Italy was in turn subject to complex colonization processes, that made its fauna biogeographically very complex, albeit not particularly rich.

**Abstract:**

As a natural bridge between Europe and Africa, Italy occupies a prominent position to understand the biogeography of Europe. The influence of climatic, spatial, and historical factors on current patterns of species richness and turnover (i.e., inter-regional biogeographical differences) has been analyzed for 88 species occurring in 17 Italian natural regions. Use of multimodel inference showed that odonate richness decreased southwards in response to decreasing rainfall, as expected for animals that depend on freshwater for their development. Use of Mantel tests indicated that patterns of inter-regional similarities were influenced by both climate and geographical distances. These patterns, as highlighted using Non-Metric Multidimensional Scaling, indicate a role for historical factors. Biogeographical similarities between Italian regions and adjacent areas revealed multiple colonization pathways. These results, coupled with the overall southward decrease in species richness, suggest that, after serving as a Pleistocene refuge from which odonates may have colonized medio-European areas, Italy was in turn subject to colonization from north to south. This resulted in Italian odonate fauna being less species rich compared to faunas in the medio-European territories, but also being biogeographically very complex.

## 1. Introduction

Odonata (dragonflies and damselflies) are a small order of predaceous insects including 6366 recognized species worldwide [1]. Due to their relatively large size and distinctive color patterns, which allow the identification of many species in the field, odonates are favored insects among professional and amateur naturalists. Odonate popularity in Europe is demonstrated, for example, by the recent increase in the number of red lists [2,3,4,5,6], field guides [7,8,9,10,11,12] and atlases [13,14] dedicated to these insects.

There are 143 species in the European odonate fauna [15,16], whose ecology and distribution are in general well known, making these insects ideal model organisms for biogeographical analyses. Notably, recent comprehensive biogeographical research on the European odonates revealed the presence of strong differences among regions due to current ecological setting and historical factors, among which Pleistocene glaciations exerted a prominent role [17,18,19,20].

Among European countries, Italy is considered of paramount biogeographical importance, because of its peculiar geographical position in the center of the Mediterranean basin and extremely intricate geological history [21,22,23,24,25,26,27]. Quite surprisingly, however, no research has been specifically devoted to the study of the biogeography of Italian odonates. Of course, understanding the biogeography of the Italian odonates does not only provide important insights into our comprehension of the biogeography of Italy, but may also offer an important contribution to our knowledge of the biogeography of Europe in general.

To fill this gap, I present here a first attempt to describe the biogeographical structure of the odonate fauna of Italy. Specifically, in this paper, I investigated the role exerted by climate and history in shaping odonate biogeographical patterns in the Italian peninsula and the adjacent major islands (Sicily, Corsica, and Sardinia). For this purpose, I considered climatic variables that can plausibly explain variations in species richness and spatial turnover (species replacement among regions) taking advantage of the additional power provided by testing multiple hypotheses simultaneously [28]. Specifically, I tested the following predictions:

Prediction 1a. Species richness increases southwards following the so-called latitudinal gradient as a result of the prominent role of southern areas as Pleistocene refugial centers for European fauna. The latitudinal gradient in species richness (i.e., the decrease in biodiversity from the Equator to the poles) is one of the most notable biodiversity patterns on Earth [29,30,31,32,33,34,35,36,37,38,39]. In Europe, a decrease in species richness with increasing latitude has been found in many hexapod groups, and it has been largely interpreted as a consequence of species responses to climatic gradients and historical factors [22,40,41,42,43,44,45,46]. In particular, Central and Northern Europe were largely recolonized after the last Pleistocene glaciation from Mediterranean refuges, leading to the present-day northward decrease in species richness. At a smaller, regional scale, this would translate in a southward increase in species richness along the Italian peninsula. Thus, if Italian odonates conform to the general latitudinal gradient, an inverse relationship between richness and latitude can be predicted.

Prediction 1b. If a peninsular region has been mainly colonized from the mainland, species richness is expected to decline from the base to the tip, as a result of increasing isolation (a phenomenon called “peninsula effect” [47,48]). If odonates mainly colonized the Italian peninsula from mainland Europe, a peninsula effect would translate in a pattern opposite to that of Prediction 1a, i.e., a southward decrease in species richness is predicted. Patterns of odonate richness in Europe may support the role of mainland Europe as a source of species for the Italian peninsula. Actually, in contrast with the aforementioned latitudinal gradient observed for a variety of taxa that decrease their richness from southern to northern Europe, odonate species richness peaks in the middle of the European territory [17,18,20].

As medio-European regions could not host a high odonate diversity during Pleistocene glaciations, it is expected that, with deglaciations, species that survived in the main European Pleistocene refuges (i.e., the Iberian, Italian, and Balkan peninsulas) moved northwards and converged in middle European areas, which thus accumulated species from different sources and in higher numbers. Then, these high-diversity areas may have acted as secondary dispersal centers, from which species moved southwards. If this hypothesis is correct, the Italian peninsula, after serving as a Pleistocene refuge from which species moved northwards, has been subsequently colonized (and recolonized) by the species that accumulated in temperate Europe. A peninsula effect is therefore expected as a result of increasing difficulties for colonizers to expand progressively far from the medio-European sources.

Prediction 2. Odonate diversity in Italy should correlate positively with rainfall. In general, organisms do not respond to latitude per se, but to a variety of factors subsumed by latitude, especially climatic gradients [49]. Thus, it is expected that climatic factors have a prominent role in explaining variation in odonate species richness [50,51]. In particular, the aforementioned “anomalous” latitudinal pattern observed for odonate richness in Europe [17,18,20] can be explained by the fact that these organisms need freshwater biotopes for their development. Although their adults are not confined to the proximity of water, the odonates are aquatic in their early stages [52,53]. Thus, freshwater availability, and hence rainfall, is expected to be an important driver of their species richness. The high abundance of precipitation might explain why medio-European areas have been so successfully colonized by odonates after deglaciation. In the case of the Italian territory, it is expected that odonate species richness declines southwards in response to decreasing rainfall. This expectation is opposite to what is observed in the Italian tenebrionid beetles, which are mainly thermo-xerophilous insects and show diversity patterns negatively correlated with precipitation [26].

Prediction 3. Species turnover is driven by differences in climatic conditions. Species composition of different areas is controlled by factors that filter the shared species pool [54]. If climatic factors are important filters in shaping odonate distributional patterns, and hence species composition, a correlation between inter-regional dissimilarities in species composition and climatic factors (such as temperature and rainfall) is expected [45], as already observed for tenebrionids in the same territory [26].

Prediction 4a. Species turnover possesses a merely positional component due to stochastic processes. Neutral hypotheses assume that species distribution is driven by stochastic population dynamics and spatially constrained dispersal [55]. Under these assumptions, a distance decay of similarity should emerge as a result of merely stochastic processes, independent from the ecological characteristics of the different areas [45]. However, differences in species dispersal abilities might have led to differences in distance decay patterns, as more mobile species might be less constrained by geographical distances.

Odonates are divided into two suborders, Zygoptera (damselflies) and Anisoptera (dragonflies), which differ markedly in morpho-functional and ecological traits [52,53,56]. In general, Zygoptera are smaller, have limited dispersal capability and tend to remain within a short distance from the site where emergence occurred, while Anisoptera are usually larger and have greater dispersal capability [53,56]. Thus, we expect that Zygoptera should particularly conform to this prediction, whereas more noise (due to higher dispersal capabilities) might make less clear the relationship between species turnover and geographical distances in the Anisoptera.

Prediction 4b. Species turnover reflects historical factors beyond the pure positional component and current ecological setting. If current species distributions have been influenced by historical (paleogeographical and paleoecological) events, it is expected to find geographically structured patterns which cannot be explained by only inter-regional climatic dissimilarity (as postulated by Prediction 3) or geographical proximity (as postulated by Prediction 4a). Additionally, patterns of biogeographical similarity are expected to highlight relationships among regions in accordance with our knowledge of the paleogeographical and paleoecological history of the study area. For the Italian territory, this has been clearly observed in tenebrionids, which have, in general, limited dispersal capabilities (most species are flightless) and hence are strongly affected by historical factors [26]. Thus, it is expected that the potential influence of historical factors will be more apparent in the Zygoptera.

Prediction 5. Similarity levels between Italian regions and adjacent countries exhibit distinct geographical patterns corresponding to colonization trajectories. Because of its central position in the Mediterranean basin, under the assumption of a postglacial recolonization from medio-European secondary centers of dispersion, the Italian territory should be intersected by multiple dispersal trajectories from adjacent areas. As a result of these faunal movements, Italian regions should exhibit geographically structured patterns of biogeographical similarities with adjacent countries. Previous research on tenebrionids (whose diversity in Europe is highest in southern countries) highlighted the role of the Italian territory as refugial center with a high level of endemicity, especially in southern regions [22,23,26]. In the case of odonates, however, almost all the species occurring in Italy are also widely distributed in the rest of Europe, and species richness peaks in medio-European areas. Thus, for the odonates, it is expected that similarities between Italian regions and European faunas should decrease from north to south, with the exception of the species occurring in Africa, for which an opposite pattern should be found (as observed for tenebrionids [26]).

More specifically, the following patterns of biogeographical similarity are expected: (1) North-Western Italian regions should exhibit high similarities with the French fauna through the Provencal area; (2) Northern Italian regions should also have high similarities with the Central European fauna, and this similarity should decrease southwards; (3) North-Eastern Italian regions should exhibit high similarities with Eastern European and Balkan territories, because of faunal exchanges through the Karst Plateau. Finally, (4) southern regions should exhibit a relatively high degree of similarity with the Northern African fauna, as a result of both the persistence of pre-Pleistocene (Tertiary) fauna and post-Pleistocene immigration of thermophilic species.

## 2. Materials and Methods

### 2.1. Data Collection

Mainland Italy is a long peninsula, aligned in a north–south direction, with its basis represented by the Alps (which connect the Italian mainland to the rest of Europe) and the tip extending in the center of the Mediterranean basin. Geographically, Italy (as intended in this paper) also includes the three adjacent major islands: Sicily, Sardinia and Corsica [26]. Sicily is very close to the Italian peninsula, from which it is separated by only 3.14 km. Corsica and Sardinia are very close to each other and share the same paleogeographical history, as they are part of the same microplate [26].

**Figure 1:**
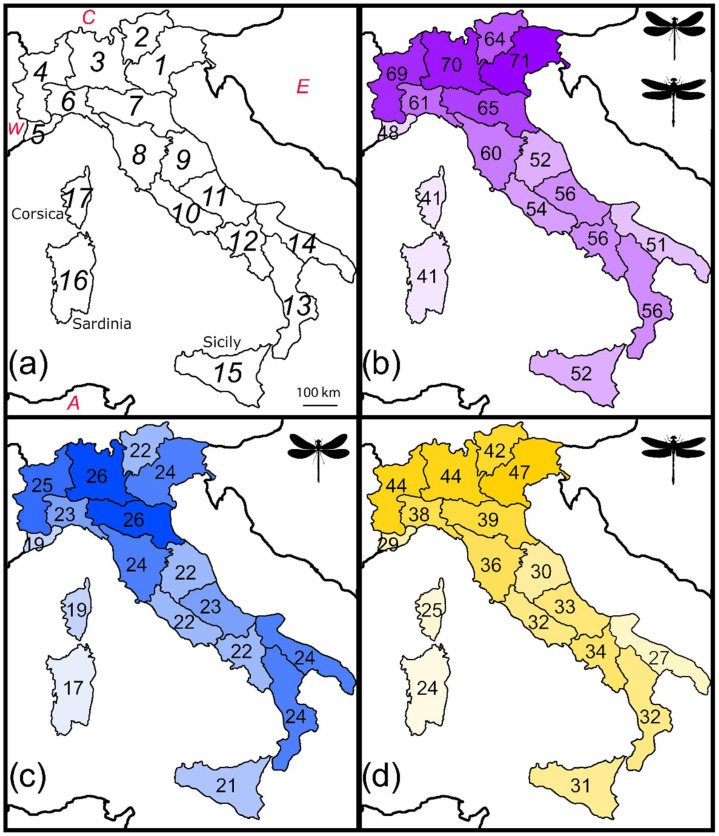
Species richness patterns of Italian odonates: (**a**) Italian natural regions (numbered from 1 to 17) and major adjacent areas (W: Western Europe; C: Central Europe; E: Eastern Europe; A: Northern Africa); (**b**) number of total odonate species in each region; (**c**) number of damselfly species (Zygoptera) in each region; (**d**) number of dragonfly species (Anisoptera) in each region.

For this study, the Italian territory was divided into 17 geographical regions (Figure 1a) as originally defined by Baroni Urbani et al. [57] and later adopted by Fattorini [26]. Use of regions, instead of grids, has been chosen to avoid problems of irregular sampling. Regional data are more accurate and comprehensive than finer-scale records [18,42,58,59,60], and can be therefore well suited to detect biogeographical patterns and construct unbiased models [18,19,61]. This type of data has been proved to be adequate to disclose the role of major environmental characteristics in determining patterns of species richness and spatial turnover [41,43,44,45,46,62,63], being robust to the violation of constant grain size [64]. Additionally, use of regions is particularly appropriate to depict species movements, because it emphasizes the influence of true natural barriers/connections among areas in determining species distributions more than arbitrarily defined squares [19].

Taxonomic treatment followed that used by the Società italiana per lo studio e la conservazione delle libellule—ODONATA.IT [65]. I did not consider subspecies because their status is usually disputed [16].

Species presence/absence in each geographical region and adjacent areas was assessed on the basis of the maps compiled by the Società italiana per lo studio e la conservazione delle libellule—ODONATA.IT [65], supplemented with information taken from Conci and Nielsen [66], D’Antonio and Utzeri [67], Boudot et al. [13], Boudot and Kalkman [14], Galliani et al. [8], Galliani et al. [9], Smallshire and Swash [11], Dijkstra and Schröter [10], and Boudot et al. [12]. I have omitted presences due to recent range expansions, but I have considered old citations of currently extinct species. Records from off-shore islands were not considered.

In total, the distribution of 88 odonate species (out of the 96 species indicated from the Italian territory [65]) was considered (Table S1).

### 2.2. Statistical Analyses

Before analysis, spatial autocorrelation in richness values was assessed using the Moran *I* index, which indicated no spatial autocorrelation (Odonata: *I* = −0.004, *p* = 0.415; Zygoptera: *I* = −0.059, *p* = 0.956; Anisoptera: *I* = 0.029, *p* = 0.209).

To test Predictions 1a and 1b, a correlation between species richness and latitude was assessed using Pearson’s correlation coefficient. In this analysis, only mainland regions were considered. Analyses were performed for the entire odonate fauna and for the two suborders (Zygoptera and Anisoptera) separately.

To evaluate the importance of climatic factors as drivers of species richness (Prediction 2), the following variables were considered for each region: total annual precipitation (Pmean), average annual temperature (Tmean), mean minimum temperature (Tmin), mean maximum temperature (Tmax) and yearly temperature diference (ΔT = Tmax−Tmin) [22,26,43,44]. Geographical and climatic data were taken from Fattorini [26].

The influence of geographical and climatic variables on species richness was investigated using a multimodel inference approach based on the corrected Akaike Information Criterion (AICc). Ordinary least squares regressions were calculated for all possible models and then all models with a ΔAICc ≤ 2 were averaged. Analyses were performed for the entire odonate fauna and for the two suborders (Zygoptera and Anisoptera) separately. To take into account possible non-linear relationships between richness and environmental variables, logarithmic transformations were tested. Since these did not change the results, linear relationships were accepted and only results with non-transformed variables will be shown here.

To test Predictions 3, 4a, and 4b, Mantel tests and partial Mantel tests between inter-regional dissimilarities in species composition and inter-regional dissimilarities in climatic factors and geographical distances were used [26]. Inter-regional faunal dissimilarities were expressed by considering the overall ß-diversity (ßsor; that is the 1-Sørensen index of similarity), the pure turnover component (ßsim; that is the 1-Simpson index of similarity) and the nestedness component (ßnest; that is ßsor-ßsim) [68,69,70]. In this respect, ßsim expressed compositional differences independently from the influence of species richness, and nestedness quantified the part of compositional change caused by ordered species loss.

To assess the influence of climate on inter-regional biogeographical similarities (Predictions 3 and 4a), ßsor, ßsim, and ßnest were correlated with matrices of inter-regional geographical distances (between centroids) and climatic distances (calculated as Euclidean distances on standardized values of climatic variables) [26]. Use of minimum distances between regional borders as an alternative measure of inter-regional geographical distances produced virtually identical results, thus only results for centroids will be presented here.

In Mantel tests, the distances between objects in a matrix A are correlated with those between the same objects in another matrix B. In partial Mantel tests, correlation between the matrices A and B is controlled for the effect of a third matrix C. In partial Mantel tests, biogeographical dissimilarities among regions were used as matrix A, while climatic and geographical distances were alternatively used as matrices B and C. Correlation between biogeographical dissimilarities (ßsor, ßsim and ßnest) and climate controlling for geographical distances was used to identify the importance of climate after removing the positional effect (Prediction 3), whereas correlation between biogeographical dissimilarities and geographical distances controlling for climate allowed the identification of the importance of positional effect (Prediction 4a) after removing the effect of climate. Analyses were performed for the entire odonate fauna and for the two suborders (Zygoptera and Anisoptera) separately.

To test the significance of the inter-regional relationships expressed by ßsor, ßsim and ßnest, the method proposed by Smith and Bermingham [71] was also applied. To this end, data were first divided into two randomly defined group of species, and ßsor, ßsim, and ßnest were calculated for the two matrices of random species. Then, for each of these three measures of dissimilarity, partial Mantel tests were conducted between the two matrices of random species, alternatively using as third matrix the geographical distances and the climatic distances. This procedure tested if there is a component of biogeographical relationships among regions that cannot be explained by current geography and climate alone (Prediction 4b). In this case, analyses were performed only for the entire odonate fauna, because the small number of Zygoptera species discouraged its partition into two random matrices.

Non-Metric Multidimensional Scaling (NMDS) was used to depict inter-regional biogeographical relationships, expressed by the Sørensen and Simpson indices. This technique depicts changes in species composition by projecting dissimilarity values among the nearest areas on a geographical map and it is therefore particularly useful to disclose multiple relationships [25,71,72]. In NMDS, Procrustes distances were used to compare solutions until a minimum stress value was reached. For the final representation, the axis with the highest variance was standardized between 0 and 1, and the second axis was rescaled according to the first one. Subsequently, the colors blue, green, yellow and red were assigned to the four corners, and RGB (red, green, blue) colors were assigned to each area according to its position in the two-dimensional graph. NMDS analyses were performed for the entire odonate fauna and for the two suborders (Zygoptera and Anisoptera) separately.

To test Prediction 5, the following areas adjacent to Italy were considered (with reference to only the closest countries): Western Europe (fauna of France), Central Europe (faunas of Austria, Switzerland, and Germany), Eastern Europe—Balkans (faunas of Slovenia, Albania, Bosnia, Herzegovina, Croatia, and mainland Greece), and Northern Africa (Tunisia). Then, the Sørensen and Simpson indices between these areas and the Italian regions were calculated and mapped, for all odonates and for the two suborders separately, using data extracted from the aforementioned literature sources [7,9,10,11,12,13,14]. Correlation with latitude was tested using Pearson’s correlation coefficient. Because spatial autocorrelation (Moran *I* values with *p* < 0.05) was detected in many instances, *p*-values of Pearson’s correlations were corrected using a modified *t*-test of spatial association.

All analyses were conducted in R 4.1.3 [73] using the following packages: ape 5.6-2 [74,75] and geosphere 1.5-14 [76] (for Moran *I* tests), SpatialPack 0.3-8196 [77,78] (for calculating the modified version of the *t*-test applied to the correlation between two spatial processes), MuMIn 1.46.0 [79] (for multimodel inference analyses), vegan 2.6-2 [80] (for Mantel tests and NMDS) and recluster 2.9 [81,82] (for NMDS).

## 3. Results

Number of total odonate species decreased along the Italian peninsula from northern regions towards the southern ones (*r* = 0.607, *p* = 0.021, Figure 1b). This was particularly true for the Anisoptera (*r* = 0.701, *p* = 0.005, Figure 1d), whereas the pattern was absent in the Zygoptera (*r* = 0.060, *p* = 0.748, Figure 1c).

The most important factors (Table 1) determining variation in species richness in Italian odonates as a whole, and for the two suborders separately, were area and total annual precipitation (Pmean).

Thus, in accordance with Prediction 1b, species richness decreased southwards, in response to decreasing rainfall, as expected according to Prediction 2.

**Figure 2:**
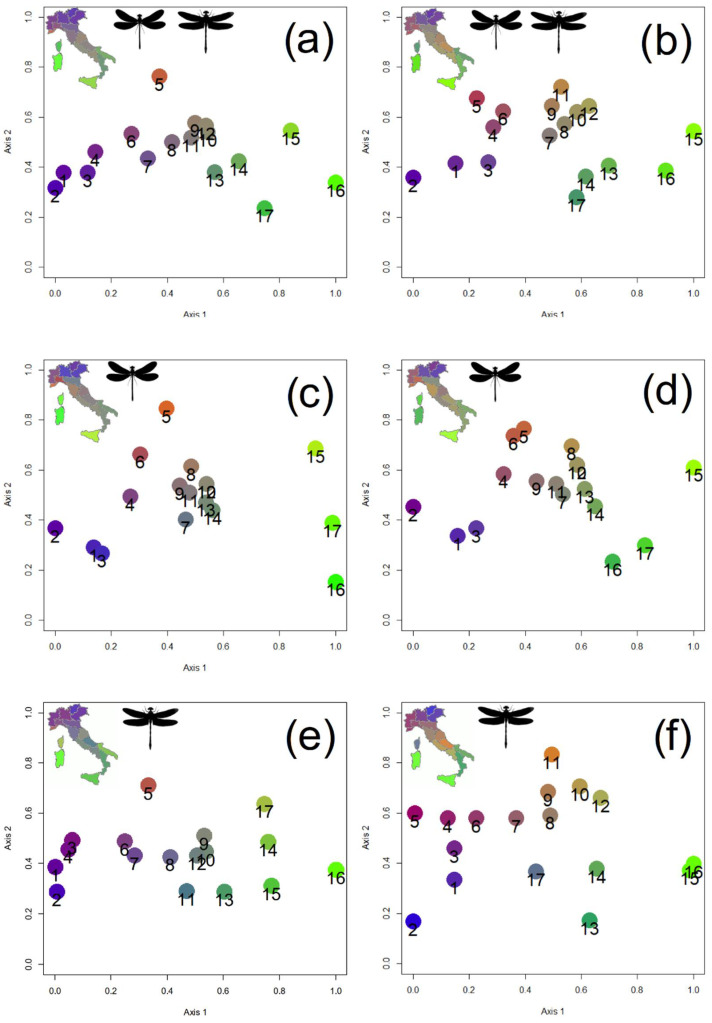
Biogeographical similarity among Italian regions expressed using Non-Metric Multidimensional Scaling (NMDS) on the Sørensen and Simpson indices based on odonate species composition: (**a**) NMDS for total odonates with the Sørensen index (Stress: 0.059); (**b**) NMDS for total odonates with the Simpson index (Stress: 0.117); (**c**) NMDS for Zygoptera with the Sørensen index (Stress: 0.058); (**d**) NMDS for Zygoptera with the Simpson index (Stress: 0.114); (**e**) NMDS for Zygoptera with the Sørensen index (Stress: 0.077); (**f**) NMDS for Zygoptera with the Simpson index (Stress: 0.116). Italian natural regions are numbered as in Figure 1a.

NMDS conducted on the entire order using the Sørensen index (Figure 2a) disclosed the following relationships among the Italian regions. First, Sicily and Sardinia (regions 15 and 16) are well apart from all the mainland regions, which, in turn, form two main groups: one including the Alpine regions (regions 1, 2, 3, and 4), and the other including the regions south of the Po River. Within the latter, Calabria and Apulia (regions 13 and 14), which are two small peninsulas, form a distinct group, placed close to Sicily and Sardinia. Corsica (region 17) lies between these two islands and peninsular Italy. Notably regions 6 and 7 occupy a transitional position between the Alpine regions and the Apennine ones. Use of the Simpson index (Figure 2b) produced a slightly different arrangement, showing a clear distinction among: (1) all regions north of the Po River (regions 1–6), (2) regions south of the Po River (regions 7–12), (3) Calabria, Apulia and Corsica, and (4) the islands of Sicily and Sardinia. These results are consistent with the paleogeographical and paleoecological history of the study area (see Discussion), thus supporting Prediction 4b.

NMDS conducted on the Zygoptera using the Sørensen (Figure 2c) and Simpson (Figure 2d) indices were similar to those achieved for the entire order, but with Corsica closer to Sicily and Sardinia, and a clear separation, within the northern regions, between eastern (1, 2 and 3) and western (4, 5 and 6) regions.

NMDS conducted on the Anisoptera using the Sørensen index (Figure 2e) were very similar to those achieved for the entire order. When the Simpson index was applied (Figure 2f), four main groups were recovered. A first group included all regions corresponding to the Alps and the Po Valley (regions 1–6). A second group included central and southern regions, from 7 to 12. A third group included Calabria, Apulia, and Corsica. Finally, Sicily and Sardinia were extremely similar and well separated from all other regions.

The Sørensen distances calculated for all species were correlated (Mantel tests) with both geographical (*r* = 0.573, *p* < 0.001) and climatic distances (*r* = 0.711, *p* < 0.001). The same results were found for both the Zygoptera (*r* = 0.416, *p* < 0.001 for geographical distances, and *r* = 0.653, *p* < 0.001 for climatic distances, respectively) and the Anisoptera (*r* = 0.618, *p* < 0.001 for geographical distances, and *r* = 0.687, *p* < 0.001 for climatic distances, respectively).

The Simpson distances calculated for all species were correlated (Mantel tests) with both geographical (*r* = 0.603, *p* < 0.001) and climatic distances (*r* = 0.68, *p* <0.001). The same results were found for both the Zygoptera (*r* = 0.432, *p* = 0.002 for geographical distances, and *r* = 0.653, *p* < 0.001 for climatic distances, respectively) and the Anisoptera (*r* = 0.655, *p* < 0.001 for geographical distances, and *r* = 0.591, *p* < 0.001 for climatic distances, respectively).

For the total number of species, nestedness was weakly correlated (Mantel tests) with geographical distances (*r* = 0.221, *p* = 0.042), and more distinctly with climatic distances (*r* = 0.352, *p* = 0.013). For the Zygoptera, nestedness was not correlated either with geographical (*r* = 0.0626, *p* = 0.271) or climatic distances (*r* = 0.143, *p* = 0.189). By contrast, for the Anisoptera, nestedness was correlated with both the geographical (*r* = 0.239, *p* = 0.018) and the climatic distances (*r* = 0.408, *p* = 0.002).

Partial Mantel tests (Table 2) revealed that correlation between biogeographical dissimilarities (expressed by the Sørensen and Simpson indices) and climate remained significant even after controlling for geographical position, and that correlation between biogeographical dissimilarities and geographical position remained significant even after controlling for climate (with the exception of the correlation of Sørensen dissimilarity with centroids after correcting for climate in the Zygoptera). This highlights the influence of climate on biogeographical dissimilarities independently from geographical position (thus supporting Prediction 3), and the influence of geographical position independently from climate (thus supporting Prediction 4a).

Use of partial Mantel tests with matrices of random species showed that biogeographical relationships expressed by the Sørensen index were very strong, even after removing the variation that resulted from their geographical position (*r* = 0.777, *p* < 0.001) or climatic similarity (*r* = 0.677, *p* < 0.001). Biogeographical relationships among regions expressed by the Simpson index were weaker, but still significant, even after removing the influence of geographical proximity (*r* = 0.416, *p* < 0.001) or climate (*r* = 0.260, *p* = 0.030). Nestedness among regions was significant, even after removing the influence of geographical proximity (*r* = 0.422, *p* = 0.002) or climate (*r* = 0.353, *p* = 0.002). This indicates that the faunal relationships are not the mere consequence of spatial arrangements, but reflect a biological history (thus supporting Prediction 4b).

**Figure 3:**
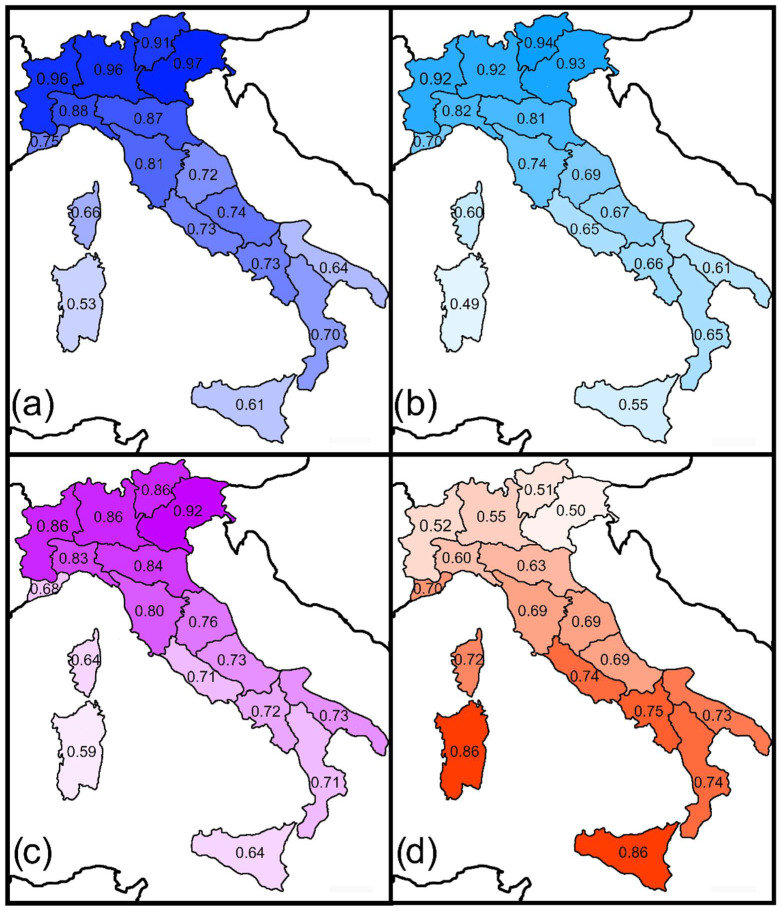
Spatial variations in the Sørensen index between Italian regions and adjacent major areas for odonates (total number of species): (**a**) Western Europe; (**b**) Central Europe; (**c**) Eastern Europe; (**d**) Northern Africa. Results for Zygoptera and Anisoptera are shown in Figures S1 and S3, respectively.

**Figure 4:**
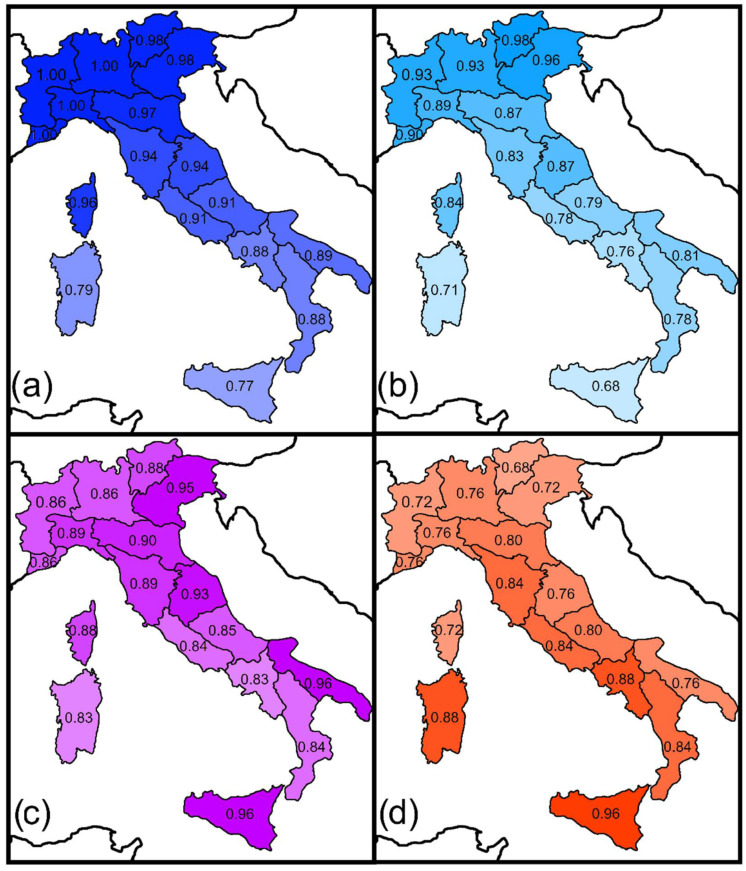
Spatial variations in the Simpson index between Italian regions and adjacent major areas for odonates (total number of species): (**a**) Western Europe; (**b**) Central Europe; (**c**) Eastern Europe; (**d**) Northern Africa. Results for Zygoptera and Anisoptera are shown in Figures S2 and S4, respectively.

Sørensen and Simpson similarities between Italian regions and adjacent areas showed distinct latitudinal trends (Figure 3 and Figure 4, Figures S1–S4, Table 3). For the Western European, Central European, and Eastern European faunas, similarities decreased southwards. By contrast, for the Northern African fauna, similarity increased southwards. These results support Prediction 5.

## 4. Discussion

In contrast to other insect groups that showed an increasing trend in diversity from northern to southern areas (such as dung beetles and tenebrionid beetles [26,83]), the species richness of Italian odonates decreased southwards.

This contrasts with Prediction 1a (latitudinal gradient), but it is consistent with a positive correlation between odonate species richness and latitude in the Western Mediterranean region [19] and the latitudinal variation observed at the European scale, where odonate species richness does not peak in the Mediterranean refuge areas, but in relatively humid Central European areas [17,18,20]. In particular, the highest values of odonate species richness in Europe have been documented in and around Alpine territories [18].

The peninsula effect (Prediction 1b) has been frequently proposed as an explanation for decreasing species richness from northern to southern regions observed for various animal groups (including birds, small mammals, carabid beetles, hydradephagan beetles and ants), along the Italian peninsula [47,84].

In the case of odonates, this can be related to their need of freshwater for oviposition and nymphal development. The decrease in odonate species richness southwards along the Italian peninsula can be therefore explained by increasing arid conditions (higher temperatures and lower rainfall) in southern regions (where the prevailing Köppen–Geiger climate type is Hot-temperate (Csa) [85]), which makes waterbodies less available. This relationship is clearly highlighted by the fact that rainfall is the most important predictor of odonate species richness in Italy (as expected according to Prediction 2) and a major driver of odonate diversity also at the European level [17].

In accordance with Prediction 3, inter-regional biogeographical relationships were correlated with climatic dissimilarities. Regions 1–3 (and 4 in some analyses), which are characterized by low temperatures and abundant rainfall (with large sectors occupied by the Köppen–Geiger climate types Cool temperate (Cf) and Cold temperate (Dw) [85]), form a distinct group with high faunal similarities. Another distinct group is represented by regions 8–12, which correspond to the regions mainly occupied by the mountain ranges of the Apennines, with higher temperatures and lower precipitations in summer months (with prevailing Köppen–Geiger climates Sub-coastal temperate (Cs) and Sub-continental temperate (Cf) [85]). Regions 5–6, which show a biogeographical transitional character between these two groups, also have intermediate bioclimatic conditions, being at the intersection between the temperate and the Mediterranean bioclimes [86] and between the Medioeuropean and the Mediterranean floristic regions in Europe [87]. Regions 13 and 14 are two small peninsulas and this relative isolation may explain why their odonate faunas are rather distinct from those of the other mainland regions, especially for the Anisoptera. The two main islands, Sicily and Sardinia, appeared very isolated in all analyses, which can be explained by both their climatic conditions (low precipitation and high temperatures [85]) and isolation. Both Sardinia and Corsica have impoverished faunas, showing the smallest number of species (both for the entire order and for the two suborders considered separately) among all Italian regions. Corsica is biogeographically well isolated, but does not show a strong similarity with Sardinia, which contrasts with its closeness to this island (with which it shares the same geological history, both belonging to the same microplate) and the high similarity in species composition between the two islands observed in butterflies [25], tenebrionids [26], carabids [57] and chrysomelids [57], but which is consistent with previous findings on odonates [19]. In particular, for the Anisoptera, when the pure turnover (the Simpson index) is considered, Corsica showed a rather high similarity with central mainland regions, which suggests that its fauna has been largely influenced by recent immigrations from Italian mainland, as already supposed by Heiser et al. [19]. It may also appear surprising the strong biogeographical isolation of Sicily, which is very close to the Italian peninsula. This biogeographical isolation is, however, consistent with previous findings on odonates [19], carabids [57] and chrysomelids [57], and in partial agreement with results obtained for butterflies [25] and tenebrionids [26]. This pattern can be explained by climatic reasons. Climate in Sicily is, in general, much warmer and more arid than in the adjacent mainland (Calabria, region 13), because mountains are less abundant in Sicily than in Calabria [85].

Mantel tests and NMDS analyses indicated that the odonate distributions in Italy are geographically strongly structured, beyond the effects of climatic conditions (Prediction 4a). For example, despite a relative uniformity of climatic conditions along all the Alpine arch, the Alpine regions tend to form two distinct groups (one including regions 1–3; the other including regions 4–6), as a consequence of the orographic subdivision of the Alps, and different colonization trajectories (see below). This is particularly apparent in the Zygoptera (which have lower dispersal capability) when the Simpson index (which is not influenced by nestedness) is used.

Some biogeographical patterns suggest a possible role of historical factors, as postulated by Prediction 4b. For example, region 14 (Apulia), which is a flat and arid region (being dominated by a Hot temperate (Csa) climate in Köppen–Geiger classification [85]), appeared to be similar, in its faunal composition, to region 13 (which is mainly mountainous and much less arid, with large sectors occupied by Köppen–Geiger Sub-coastal temperate (Cs) and Sub-continental temperate (Cf) climates [85]). Interestingly, these two regions appeared also similar (with Baroni Urbani and Buser coefficient of similarity) for carabid and chrysomelid beetles [57]. During the Pleistocene glaciations, these two regions were environmentally and climatically similar [88,89], which suggests that their current faunal similarity may be partly due to a shared paleoecological history. Notably, the biogeographical proximity between these two regions appeared higher in the Zygoptera, which have lower dispersal capabilities.

According to Heiser and Schmitt [18], Italy shows a high impact of the generally widespread European elements and biogeographical links with Balkans. This is confirmed and further clarified by the analyses presented in this paper. In accordance with Prediction 5, geographically structured patterns of biogeographical similarities between Italian regions and adjacent countries were detected, with two opposite trajectories of similarities: one with European territories (with similarity decreasing southwards), and the other with Northern Africa (with similarity decreasing northwards). In particular, the Simpson index revealed a strong similarity of the Western Alpine regions with the Western European fauna, whereas the Eastern Alpine regions showed a strong similarity with the Eastern European fauna. Notably, values of similarity between Italian regions and Eastern Europe were consistently higher in the Adriatic regions than in the Tyrrhenian ones, indicating that exchanges occurred along territories east of the Apennines, which acted as a substantial barrier preventing wide exchanges with the Tyrrhenian side. Similarity with the Central European fauna was rather uniformly high along the whole Alpine chain and decreased southwards. These colonization pathways are consistent with multiple dispersal trajectories hypothesized for tenebrionid [26] and cholevine [90] beetles. Corsica appeared to show higher values of similarity with Western, Central and Eastern European faunas than with that of Sardinia, which further supports that its fauna was widely influenced by recent colonization from the Italian mainland, as already suggested by Heiser et al. [19]. By contrast, Sardinia has a strong similarity with the African fauna, which is consistent with the fact that this region is particularly prone to be colonized by African species, as testified by the increasing number of African odonates that reached the island recently [91], probably because of particularly favorable climatic conditions.

In general, biogeographical relationships appeared stronger for the Zygoptera than for the Anisoptera, which is in contrast with conclusions reached by Heiser and Schmitt [18], but confirms previous research illustrating a higher tendency of the Zygoptera in producing clearly structured biogeographical patterns [16,92,93,94,95], possibly as a reflection of their weaker dispersal ability as compared to the Anisoptera [20].

## 5. Conclusions

Biogeographical patterns shown by Italian odonates suggest that their distribution has been largely affected by faunal movements in glacial-interglacial phases. During Pleistocene glaciations, most European fauna retreated into southern refugial areas (mainly the Iberian, Italian and Balkan peninsulas). After deglaciation, Central and Northern European areas were recolonized from these southern refuges. The highest odonate diversity recorded in Central Europe may be explained by the persistence of abundant freshwater biotopes because of high rainfall. The faunal impoverishment observed southwards in the odonate fauna along the Italian peninsula suggests that the Italian fauna was largely shaped by postglacial immigration from Central European areas. In other words, with deglaciation, odonates first moved from southern areas to Central Europe, and then from Central Europe to southern areas. The southward impoverishment in odonate species richness is a result of both the peninsula effect (colonization is more difficult in more distant areas) and the filtering effects of climatic factors (increasing aridity in southern areas). The Italian fauna is not very rich in species, but is biogeographically complex, and current similarities with adjacent regions suggest that the territory was colonized through multiple trajectories.

## Figures and Tables

**Table 1:** Results of multimodel selection for the influence of geographical and climatic variables on odonate richness in Italy. For the total Odonata and Anisoptera, model-averaged coefficients (full average) are given; for the Zygoptera only one model was selected on the basis of AICc values. *p*-values are calculated as probability > |z| for the model-averaged coefficients of total Odonata and Anisoptera, and as probability > |*t*| for the single model obtained for Zygoptera. SE: standard error; sw: sum of weights; Pmean: total annual precipitation; Tmean: average annual temperature; Tmin: mean minimum temperature; Tmax: mean maximum temperature.

	Total Odonata				Zygoptera			Anisoptera			
	Estimate	SE	*p*-Value	sw	Estimate	SE	*p*-Value	Estimate	SE	*p*-Value	sw
**Intercept**	−9.791	34.717	0.782		7.758	3.188	0.029	−7.515	30.226	0.806	
**Area**	0.001	<0.0001	<0.00001	1	1.921 × 10^−4^	5.991 × 10^−5^	0.006	4.578 × 10^−4^	1.132 × 10^−4^	<0.001	1
**Pmean**	0.049	0.011	<0.00001	1	0.015	0.004	0.001	0.034	0.009	<0.001	1
**Latitude**	0.547	0.723	0.459	0.42				0.347	0.598	0.568	0.28
**Tmean**	−0.208	0.422	0.629	0.23				−0.312	0.474	0.516	0.34
**Tmin**	−0.148	0.362	0.689	0.17				−0.179	0.382	0.645	0.20
**Tmax**	−0.134	0.329	0.692	0.17				−0.144	0.331	0.668	0.18

**Table 2:** Partial Mantel tests of biogeographical distances against climatic and geographical distances for Italian odonates.

Matrix Correlation		Biogeographical Distances					
Matrix A × Matrix B	Matrix C (Controlling)	Sørensen (βsor)		Simpson (βsim)		Nestedness (βnest)	
		*r*	*p*	*r*	*p*	*r*	*p*
**Odonata**							
Climatic distances	Centroids	0.620	<0.001	0.580	<0.001	0.290	0.038
Centroids	Climatic distances	0.404	<0.001	0.454	<0.001	0.075	0.246
**Zygoptera**							
Climatic distances	Centroids	0.574	0.002	0.569	<0.001	0.129	0.207
Centroids	Climatic distances	0.182	0.105	0.207	0.050	−0.002	0.456
**Anisoptera**							
Climatic distances	Centroids	0.583	<0.001	0.440	0.003	0.346	0.003
Centroids	Climatic distances	0.478	0.002	0.541	<0.001	0.069	0.296

**Table 3:** Correlation between biogeographical similarity (the Sørensen and Simpson indices) and latitude for species living also in Western Europe, Central Europe, Eastern Europe, and Northern Africa.

	Sørensen Index		Simpson Index	
	*r*	*p*	*r*	*p*
**Total Odonata**				
Western Europe	0.86	0.025	0.921	0.24
Central Europe	0.881	0.019	0.950	0.009
Eastern Europe	0.791	0.025	0.495	0.066
Northern Africa	−0.896	0.015	−0.804	0.041
**Zygoptera**				
Western Europe	0.469	0.088	0.404	0.108
Central Europe	0.698	0.029	0.803	0.019
Eastern Europe	0.635	0.039	0.768	0.023
Northern Africa	−0.789	0.023	−0.611	0.09
**Anisoptera**				
Western Europe	0.860	0.025	0.921	0.024
Central Europe	0.881	0.019	0.951	0.009
Eastern Europe	0.791	0.025	0.495	0.066
Northern Africa	−0.896	0.015	−0.804	0.041

## Data Availability

Species distribution data are given in Table S1. Geographical and climatic data are taken from Fattorini [18].

## Supplementary Materials

The following supporting information can be downloaded at: https://www.mdpi.com/article/10.3390/biology11060886/s1, Figure S1: Spatial variations in the Sørensen index between Italian regions and adjacent major areas for Zygoptera; Figure S2: Spatial variations in the Simpson index between Italian regions and adjacent major areas for Zygoptera; Figure S3: Spatial variations in the Sørensen index between Italian regions and adjacent major areas for Anisoptera; Figure S4: Spatial variations in the Simpson index between Italian regions and adjacent major areas for Anisoptera; Table S1: Species distribution.
