# Supplementary material for: Odonate Diversity Patterns in Italy Disclose Intricate Colonization Pathways
Source: Biology (Basel). 2022 Jun 8;11(6):886. doi: 10.3390/biology11060886 (PMC9220287; doi:10.3390/biology11060886)
Supplement: Supplementary file 1 [file biology-11-00886-s001.zip › Figure S1.pdf]

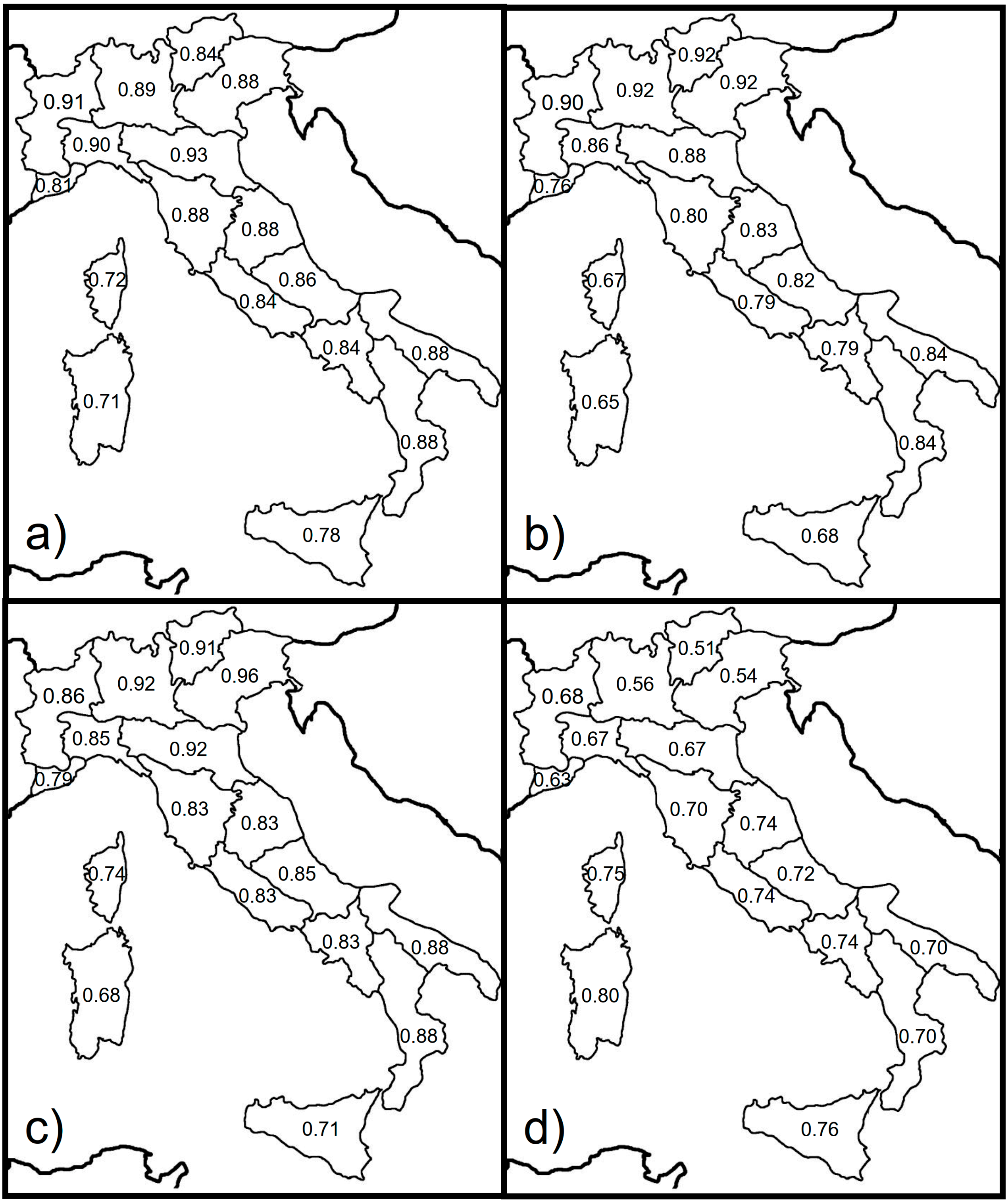

Figure S1. Spatial variations in the Sørensen index between Italian regions and adjacent major areas for Zygoptera: (**a**) Western Europe; (**b**) Central Europe; (**c**) Eastern Europe; (**d**) Northern Africa.
